# Treatment Response Distinguishes Persistent Type of Methamphetamine Psychosis From Schizophrenia Spectrum Disorder Among Inmates at Japanese Medical Prison

**DOI:** 10.3389/fpsyt.2021.629315

**Published:** 2021-07-19

**Authors:** Yosuke Sekiguchi, Takayuki Okada, Yusuke Okumura

**Affiliations:** ^1^Department of Psychiatry and Behavioral Sciences, Graduate School of Medical and Dental Sciences, Tokyo Medical and Dental University, Tokyo, Japan; ^2^Medical Correction Center in East Japan, Tokyo, Japan

**Keywords:** methamphetamine associated psychosis, schizophrenia spectrum disorder, chlorpromazine equivalent dose, medical prison, antipsychotics

## Abstract

**Introduction:** Persistent methamphetamine-associated psychosis (pMAP) is a disorder similar to schizophrenia, so much so that the differences in clinical symptoms and treatment response between the two remain unknown. In this study, we compared the features of pMAP with those of schizophrenia spectrum disorders (SSD).

**Materials and Methods:** This was a retrospective quasi-experimental case-control study of inmates in a medical prison. The behavioral problems, clinical symptoms, and chlorpromazine (CP)-equivalent doses of 24 patients with pMAP and 27 with SSD were compared.

**Results:** Patients in the pMAP group were hospitalized for fewer days than those in the SSD group (281.5 vs. 509.5; *p* = 0.012), but there were no other significant group differences in behavioral problems or clinical symptoms. The pMAP group received fewer antipsychotics in CP-equivalent doses than the SSD group at 4, 8, and 12 weeks after admission and at the time of discharge (*p* = 0.018, 0.001, 0.007, and 0.023, respectively). The number of CP-equivalent doses in the SSD group tended to increase after admission, but not in the pMAP group.

**Discussion:** These findings suggest that differentiation between pMAP and SSD based on behavior and symptoms alone may be difficult, and that patients with pMAP may respond better to treatment with a lower dose of antipsychotic medication than those with SSD. Further confirmatory studies are warranted.

## Introduction

It is estimated that 4.96 million people use amphetamine-type stimulants (1). Methamphetamine is one such amphetamine-type stimulant and a highly potent drug closely linked to violent crime (2), recidivism (3), and drug crimes. This leads to confusion in clinical settings, especially in forensic or correctional medical settings, when it comes to diagnosis and treatment of patients with psychotic symptoms suspected of using methamphetamine. As such, psychosis induced by methamphetamine (methamphetamine associated psychosis, MAP) has received recent attention (4, 5). Symptoms of MAP include hallucinations, delusions, negative symptoms, and cognitive impairment, which are similar to those of schizophrenia (6–9). Moreover, psychiatrists use antipsychotics to treat both MAP and schizophrenia (9, 10). With regard to symptomatology and pharmacotherapy, it is difficult to differentiate MAP from schizophrenia spectrum disorder (SSD). In Japan, due to the occurrence of MAP associated with the period of methamphetamine abuse in the mid-20th century, it has been regarded as a different disease from schizophrenia, based on the discovery of differences through clinical observation. As described in the review by Yui et al. (10), there was a history of being viewed as a different disease, with several differentiating features in the symptoms and course of the disease, but because many papers were written in Japanese, the impact on the global academic community was limited.

The diagnosis of MAP is currently based on the Diagnostic and Statistical Manual of Mental Disorders 5th edition (DSM-5) criteria for substance-induced psychosis (11). This requires an individual to present with either delusions or hallucinations that abate within ~1 month of drug cessation. However, some researchers, especially in Japan, characterize MAP as a psychotic state that occurs as a result of methamphetamine dependence, broken into two types; “transient” and “persistent” (12, 13). The transient type presents with either delusions or hallucinations that abate within ~1 month of drug cessation, while the persistent type can cause delusions and hallucinations for months or years after drug cessation.

Similar to previous Japanese studies, a recent review (14) divided MAP into two types: “acute” (corresponding to “transient”) and “persistent.” However, few studies have distinguished between acute and persistent MAP (4). We assumed the difficulties in distinguishing MAP and SSD are rooted in confusion between acute and persistent types. For instance, McKetin et al. (15) classified methamphetamine-induced transient psychosis and persistent psychosis and compared them to primary psychosis. However, they excluded patients from the MAP group who met the DSM criteria for schizophrenia. For this reason, psychosis due to methamphetamine that caused schizophrenia was not compared, and the qualitative difference not mentioned. Moreover, studies that investigated MAP symptoms had several limitations. Most of them considered abstinence from drugs, but the information was derived from self-report data, indicating the reliability of drug-cessation data was fragile. To overcome these limitations, we chose to include only patients in a medical prison. This makes our drug-cessation data more reliable; Japanese prisons enforce strict rules banning the use of illegal drugs. To some extent, the criminal tendencies of patients in the MAP and SSD groups in our sample could be considered more similar than those of patients with MAP and SSD in the general population. In other words, when the general population is included in such a study, most of the people in the SSD group will be non-criminals, while the MAP group will show some kind of deviant behavior, and thus there will be a large difference in criminal tendency between the two groups. However, since all the individuals in our sample had committed some type of crime, we did not expect to see a significant difference in criminal tendencies between groups compared to the general population.

We hypothesized that contrary to the DSM-5 definition, persistent MAP (pMAP) and SSD are separate disorders. In this study, we focused on pMAP and chronic SSD to clarify the differences between them with respect to life history, behavioral problems, clinical symptoms, and response to pharmacotherapy.

## Materials and Methods

### Study Design and Participants

We conducted a retrospective quasi-experimental case-control study using structured prison and medical records of patients in a medical prison. Included patients were transferred from general prisons to psychiatric wards in the Medical Correction Center in East Japan, formerly known as Hachioji Medical Prison Hospital, for psychiatric treatment from April 2010 to July 2020. Hachioji Medical Prison Hospital was one of four hospitals in Japan with the ability to provide inpatient treatment for inmates, mainly in the medical, surgical and psychiatric wards. In January 2018, Hachioji Medical Prison Hospital was moved to the Medical Correction Center in East Japan. We used the number of days to discharge as one of the indices for treatment response; therefore we only included patients discharged until November 2020. We investigated the data of 129 admitted patients originally diagnosed with schizophrenia, schizoaffective disorder, MAP, or drug-induced psychosis who also experienced subsequent complications due to methamphetamine use. We defined patients with pMAP as those (1) who met the DSM-5 criteria for schizophrenia or schizoaffective disorder, (2) who had a history of multiple instances of methamphetamine use, and (3) whose onset of psychosis was followed by methamphetamine use. To ensure we included only patients with precise diagnoses free of ambiguity, we excluded two patients who could have been classified into the SSD group because they had histories of methamphetamine use prior to onset of psychosis. Sufficient information was available to correctly diagnose and categorize 51 patients.

These 51 patients were classified into two groups: the MAP group (*n* = 24) and the SSD group (*n* = 27). None of the patients had used amphetamine or dextroamphetamine.

We collected data on diagnosis, life history, medical history, behavioral problems, and pharmacotherapy from the medical records.

### Diagnosis and Measures Assessing Life History

Psychiatric disorders and comorbidities were diagnosed based on the DSM-5 criteria. Original diagnoses recorded in the medical records were made by attending doctors during the hospital stay, and an experienced psychiatrist (YS) confirmed the accuracy of the diagnoses using data from the medical records and excluded patients who did not meet the diagnostic criteria for pMAP or SSD.

The pMAP and SSD groups were compared with respect to age, gender, race, years of illness, years of methamphetamine use, years from methamphetamine use to onset, estimated intelligence quotient [assessed using a test called CAPAS (16) from the Ministry of Justice], medical history (whether treatment continued until change to outpatient status/hospitalization/arrest), suicide attempt, comorbidity, educational background, family history during childhood (familial antisociality, poverty, divorced/bereaved parents, psychiatric family history, childhood abuse), school refusal, delinquency, regular work experience, marriage/divorce history, crime type, first crime/repeated crime, abuse of other drugs (thinner or cannabis).

### Clinical Observations

First, we examined the number of hospitalization days at the medical prison. Regarding behavioral problems after admission, we examined yelling, self-harm, verbal abuse, physical violence, food refusal, and playing with one's own feces. We also examined auditory hallucination, visual hallucination, tactile hallucination, persecutory delusion, disorganized speech, manic state, and lack of insight. Data were recorded by nurses based on their observations within a strict 24-h surveillance period inside the medical prison. These patterns of behavior were summarized using binominal values: 1, existence; 0, absence.

We considered the following variables for pharmacotherapy: antipsychotic doses before admission, 4, 8, and 12 weeks after admission, and at time of discharge. The pharmaceutical data was compared using chlorpromazine (CP)-equivalent dose conversion (17). As two patients were hospitalized <12 weeks, we assumed their doses at 12 weeks were same as those upon discharge.

### Statistical Analysis

Continuous data were analyzed using the Mann-Whitney *U*-test. Categorical data were analyzed using Fisher's exact test. All tests were two-sided, with significance set at *p* < 0.05. The false discovery rate [Benjamini-Hochberg procedure (18)] was used to correct *p*-values for multiple testing of CP-equivalent doses at each time point between groups. To compare the CP-equivalent doses within groups at each time point, we used Friedman's test and Scheffe's *post hoc* test. All analyses were conducted using BellCurve for Excel (Social Survey Research Information Co., Ltd. Tokyo, Japan).

### Ethical Approval

We conducted this research following the principles outlined in the Declaration of Helsinki. This study was a retrospective study without utilizing any specimens and the information utilized in the research had been anonymized. The need for informed consent was waived in accordance with Ethical Guidelines for Medical and Health Research Involving Human Subjects in Japan. This study was reviewed and approved by the Clinical Research Ethics Board of the Medical Correction Center in East Japan.

## Results

### Sample Characteristics

The total sample consisted of 51 participants with a mean age of 39.8 years [standard deviation (SD) 11.9]; 84.3% were male, and 98.0% were Japanese. All patients had a psychotic disorder: schizophrenia, schizoaffective disorder, MAP, drug-induced psychosis, or residual or late-onset psychotic disorder induced by MA use. In this sample, pMAP occurred in 47.1% of the sample and SSD occurred in 52.9%.

### Background Characteristics

The pMAP group experienced a shorter duration of psychotic disorder (10.0 vs. 15.7 years, *p* = 0.049) and had a higher estimated intelligence quotient (77.5 vs. 64.2, *p* = 0.020) compared to the SSD group. The pMAP group had a history of cannabis use (27.5 vs. 5.9%, *p* = 0.001), more incarceration due to stimulant control law violations (62.5 vs. 0.0%, *p* < 0.001), and increased delinquency in childhood (79.2 vs. 25.9%, *p* < 0.001). No significant differences were found for other variables, including age and sex (Table 1).

**Table 1:** Background characteristics of pMAP and SSD patients in the medical prison.

	**pMAP (*****n*** **= 24)**		**SSD (*****n*** **= 27)**			***U*-test**
	**Mean**	**SD**	**Mean**	**SD**	***p*-value**	
Age at admission	38.3	13.1	41.1	10.7	0.143	
Age at onset	28.2	7.9	25.4	8.3	0.261	
Years of psychotic disorder	10.0	10.7	15.7	13.1	0.049	^*^
Age at first MA use	20.7	4.0	–	–		
Years between first MA use and onset	7.5	5.9	–	–		
Estimated intelligence quotient	77.5	18.9	64.2	20.0	0.020	^*^
	**Number**	**%**	**Number**	**%**		**Fisher's exact test**
Sex					0.127	
Male	18	75.0	25	92.6		
Female	6	25.0	2	7.4		
Admission to correctional facilities					0.265	
First time	12	50.0	18	66.7		
Multiple times	12	50.0	9	33.3		
**Other drug abuse**						
Thinner	11	21.6	6	11.8	0.136	
Cannabis	14	27.5	3	5.9	0.001	^**^
**Type of crime**						
Stimulants Control Law	15	62.5	0	0.0	0.000	^**^
**Childhood experience**						
Maltreatment	5	22.7	7	26.9	1.000	
Poverty	6	30.0	8	34.8	1.000	
Divorce or bereavement of parents	12	50.0	10	38.5	0.569	
Antisocial family members	3	13.6	1	3.7	0.314	
Bullied	2	13.3	5	22.7	0.677	
School refusal	8	50.0	6	27.3	0.187	
Delinquency	19	79.2	7	25.9	0.000	^**^
**Education level**						
Less than high school diploma	18	75.0	13	50.0	0.086	
Work experience for >6 months	16	69.6	18	66.7	1.000	
Homelessness	4	16.7	6	22.2	0.731	
Marriage history	4	16.7	2	7.4	0.402	
Suicidal behavior	15	62.5	9	33.3	0.051	
History of psychiatric treatment	21	87.5	25	92.6	0.656	
Under psychiatric treatment before arrest	11	52.4	9	36.0	0.372	
History of hospital admission	15	65.2	21	80.8	0.332	

** *p < 0.01*;

* *p < 0.05. pMAP, persistent methamphetamine-associated psychosis; SSD, schizophrenia spectrum disorders; MA, methamphetamine; SD, standard deviation*.

Both groups were more likely to include individuals who had not completed high school (75.0 vs. 50.0%), had work experience >6 months (69.6 vs. 66.7%), and were less likely to have been married (16.7 vs. 7.4%). All participants with a marriage history (*n* = 6) were divorced and single at the time of the study. Suicidal behavior was, to an extent, common in both groups (62.5 vs. 33.3%). Both groups were more likely to report history of psychiatric treatment (87.5 vs. 92.6%) and history of hospital admission (65.2 vs. 80.8%), but under psychiatric treatment before arrest was much lower than expected from treatment history (52.4 vs. 36.0%).

### Behavioral Problems and Clinical Symptoms

We found that the pMAP group had shorter hospitalizations (281.5 vs. 509.5 days, *p* = 0.012). There were no other significant group differences in behavioral problems or clinical symptoms (Table 2). More than half of patients in both groups exhibited yelling (62.5 vs. 85.2%), auditory hallucination (83.3 vs. 96.3%), persecutory delusion (75.0 vs. 85.2%), or lack of insight (75.0 vs. 85.2%).

**Table 2:** Behavioral problems and clinical symptoms of pMAP and SSD patients in the medical prison.

	**pMAP (*****n*** **= 24)**		**SSD (*****n*** **= 27)**		
	**Mean**	**SD**	**Mean**	**SD**	***p*-value**
Days of hospitalization in the medical prison	281.5	187.0	509.5	363.0	0.012^*^
	**Number**	**%**	**Number**	**%**	***p*****-value**
Yelling	15	62.5	23	85.2	0.107
Self-harm	5	20.8	4	14.8	0.718
Verbal abuse	12	50.0	15	55.6	0.782
Physical violence	2	8.3	6	22.2	0.255
Refusal of food	4	16.7	6	22.2	0.731
Playing with one's own feces	3	12.5	4	14.8	1.000
Auditory hallucination	20	83.3	26	96.3	0.175
Visual hallucination	6	25.0	8	29.6	0.762
Tactile hallucination	5	20.8	7	25.9	0.749
Persecutory delusion	18	75.0	23	85.2	0.485
Disorganized speech	9	37.5	17	63.0	0.095
Manic state	1	4.2	5	18.5	0.195
Lack of insight	17	70.8	23	85.2	0.310

* *p < 0.05. pMAP, persistent methamphetamine-associated psychosis; SSD, schizophrenia spectrum disorders; SD, standard deviation*.

### CP Equivalent Doses

**Figure 1:**
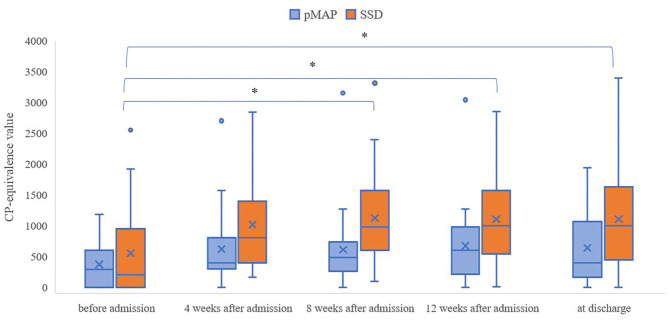
Changes in CP-equivalence value over time within the pMAP and SSD groups. Friedman test: *p* = 0.0003 in the SSD group, *p* = 0.337 in the pMAPgroup. Comparisons between time points were performed using Scheffe's *post hoc* test. pMAP, persistent methamphetamine-associated psychosis; SSD, schizophrenia spectrum disorders; CP, chlorpromazine. *p < 0.05.

We found that the pMAP group received fewer antipsychotics in CP-equivalent doses than the SSD group at 4, 8, and 12 weeks after admission and at the time of discharge (*p* = 0.018, 0.001, 0.007, and 0.023, respectively; Table 3). Friedman's test revealed a significant difference in the SSD group (*p* < 0.001), but not in the pMAP group (*p* = 0.337). In the SSD group, Scheffe's *post hoc* test revealed that CP-equivalent doses at 8 and 12 weeks after admission and at the time of discharge were higher than those before admission (*p* = 0.002, 0.012, and 0.017, respectively; Table 4). The number of CP-equivalent doses in the SSD group tended to increase after admission, which was not the case in the pMAP group (Figure 1).

**Table 3:** CP-equivalence value of pMAP and SSD patients in the medical prison.

	**pMAP** ***(n*** **= 24)**			**SSD (*****n*** **= 27)**				
**CP equivalence**	**Median**	**Q**	**Range**	**Median**	**Q**	**Range**	**z score**	***p*-value**
Before admission	290.0	(0, 600)	0–1,183	300.0	(0, 1,025)	0–2,553	0.851	0.395
4 weeks after admission	400.0	(300, 802.5)	0–2,702	803.0	(515, 1,400)	167–2,842	2.371	0.018^*^
8 weeks after admission	488.5	(287.5, 663)	0–3,156	1,000.0	(603, 1,583.5)	100–3,312	3.239	0.001^*^
12 weeks after admission	600.0	(287.5, 970)	0–3,042	1,000.0	(600, 1,579)	12.5–2,850	2.682	0.007^*^
At discharge	466.5	(275, 940.5)	0–1,936	1,000.0	(500, 1,629)	0–3,394	2.265	0.023^*^

* *p < 0.05. Q, 25% percentile, 75% percentile. After Mann-Whitney U-test, false discovery rate method (Benjamini-Hochberg procedure) was used*.

*pMAP, persistent methamphetamine-associated psychosis; SSD, schizophrenia spectrum disorders; CP, chlorpromazine*.

**Table 4:** Changes of CP-equivalence value of pMAP and SSD over time.

				**SSD (*n* = 27)**	
**CP-equivalence**	***n***	***chi square***	***df***	***p*-value**	**Friedman test**
pMAP	24	20.971	4	0.337	
SSD	27	4.545	4	0.0003	^**^
SSD					Scheffe's *post hoc* test
Before admission	4 w after admission	7.720	4	0.102	
	8 w after admission	16.434	4	0.002	^**^
	12 w after admission	12.948	4	0.012	^*^
	At discharge	11.983	4	0.017	^*^
4 w after admission	8 w after admission	1.627	4	0.804	
	12 w after admission	0.672	4	0.955	
	At discharge	0.467	4	0.977	
8 w after admission	12 w after admission	0.207	4	0.995	
	At discharge	0.351	4	0.986	
12 w after admission	At discharge	0.019	4	1.000	

* *p < 0.05*,

** *p < 0.01. CP, chlorpromazine; pMAP, persistent methamphetamine-associated psychosis; SSD, schizophrenia spectrum disorders; df, degree of freedom*.

## Discussion

To our knowledge, this is the first study to compare the symptoms and response to drug treatment of patients with pMAP and SSD in a medical prison. We found that the time of psychotic disorder was longer in the SSD group than in the pMAP group. As pMAP is not a spontaneous outbreak but rather an artificially generated psychosis caused by drug use, the onset of pMAP occurred when the patient was old enough to use drugs. For this reason, the duration of psychosis may have been shorter than that of non-artificially generated SSDs. However, as we did not find a significant difference in time of onset, no definite conclusions can be drawn from the present findings. This is a point that warrants further study. The difference in the estimated intelligence quotient is reasonable, as recent studies have shown that ~70% of schizophrenic patients show a lower intelligence quotient after disease onset (19). On the other hand, there is little research at this stage on the decline of intelligence quotient in pMAP. Perhaps pMAP does not have a significant intelligence quotient decline due to the disease. In other words, patients with schizophrenia are genetically predisposed to have pre-existing cognitive impairment, which may be associated with a post-onset decline in intelligence quotient (19), whereas pMAP is an artificial onset due to drug use, making an innate pre-existing cognitive impairment unlikely, and therefore it may be associated with a decline in intelligence quotient. It is possible that this is not the case in the general population. However, unlike the general population, the estimated intelligence quotient for the entire prison population is ~80 (16), and this study was conducted on hospitalized patients with high levels of illness; therefore, the results cannot be generalized to the community. In addition, the CAPAS itself, which is measured as the “estimated intelligence quotient,” is only a surrogate measure and should be confirmed through more detailed investigation in the future.

Significant differences in other background characteristics such as history of cannabis use, incarceration for stimulant control law violation, and childhood delinquency, can be explained by the fact that the concept of pMAP itself is closely related to the crime of stimulant control law violation, which is related to delinquency and other drug use. The concept of SSD, on the other hand, is not related to drug crime by nature. The poor educational background and poor marital status which were common to both groups may be associated with poor social adjustment before arrest and difficulties in social adjustment after release. Low treatment continuation rates at the time of arrest may also indicate a need for special treatment attention for offenders with psychotic symptoms, which warrants further study.

No significant differences were found in behavioral problems or clinical symptoms, and the common prevalence of yelling, auditory hallucinations, persecutory delusions, and lack of awareness suggests that differentiation between pMAP and SSD based on behavior and symptoms alone may be difficult. Previous studies have not reached a consensus on the differences in symptom profiles (20–24). However, as Srisurapanont et al. suggested (22), we do not believe there are behavioral or symptom profiles specific enough to predict whether a patient should be diagnosed with pMAP or SSD. From this point of view, it seems reasonable to not distinguish pMAP from schizophrenia in the DSM, which classifies disorders based on observable symptoms. In fact, a diagnostic transition from substance-induced psychosis to schizophrenia is not an uncommon phenomenon (25, 26). A recent meta-analysis showed that amphetamine-induced psychosis (and not only methamphetamine) leads to a later diagnosis of schizophrenia in ~22% of patients (24). However, this result does not indicate that substance-induced psychosis naturally morphed into schizophrenia.

The idea of regarding the two diseases as the same is not valid because the etiologies clearly differ, as should the inferred pathogenic mechanisms in the brain. Since SSD itself can be regarded as a heterogeneous entity (27–29), it will be subdivided based on its pathogenic mechanisms in the future. SSD and pMAP, which may have different etiologies, should be distinguished from each other. For example, schizophrenia with enhanced carbonyl stress (27, 28) should be differentiated from N-methylD-aspartate glutamate receptor encephalitis (29). Our view is supported by the differences in response to antipsychotic medication. In the present study, the SSD group received an increase in antipsychotic medication after admission, whereas the doses given to the pMAP group did not significantly increase; the pMAP group received significantly less antipsychotic medication after admission than the SSD group. In addition, the pMAP group had a shorter hospital stay than the SSD group. These findings suggest that the pMAP group improved and responded better to treatment than the SSD group, even with lower doses of antipsychotic medication. This is consistent with the opinion that “minimal psychotropic doses are desirable and should be combined with psychosocial interventions (21).” However, it is unclear in this study whether minimal adjustments of the antipsychotic dosage resolved symptoms or whether the improvements were due to adjustments to the type of medication. Additionally, a previous study has also shown that patients with treatment-resistant schizophrenia or other psychosis show lower verbal intelligence and fluency than treatment responders (30), and we cannot rule out the possibility that the difference in estimated intelligence quotient between pMAP and SSD in this study may have affected the outcome of treatment responses.

This study has several limitations. First, it was a retrospective study, and causality was unknown. Further research is needed before definite conclusions can be drawn. Second, the association between sex and MAP symptoms needs to be examined, as previous studies have suggested there may be sex differences (21). Third, although the present study revealed a temporal change in antipsychotic medication dose, it provided only collateral evidence of treatment responsiveness. Future studies are therefore needed to elucidate the relationship between treatment-related symptom changes and antipsychotic dosage. Fourth, the study was conducted in a medical prison, a facility that attracts the most severely mentally ill of those being sentenced; our sample did not include the less severely ill or patients in the community. Therefore, we believe there is a limit to the extent to which our findings can be applied to the general population. Fifth, this study did not use a measure of personality disorders, that could have a crucial role in symptoms managing, as well as in violence risk identification. In addition, the number of patients surveyed was relatively small, and the statistical power of the study may have been low. We used false discovery rate method for statistical correction in CP equivalent doses, but no statistical correction was used in background characteristics, behavioral problems, and clinical symptoms. Many comparisons for a sample of this size raises the possibility of Type 1 error in these sections. For these reasons, large-scale surveys are warranted.

Despite these methodological problems, the present study is important for suggesting a difference in treatment response between pMAP and SSD. In the clinical settings, pMAP would be more likely to be treated with maintenance antipsychotic medication than transient MAP; however, it would require a lower dose of antipsychotic medication than SSD, and it would be less likely to cause side effects. Minimal antipsychotic treatment should be used to reduce the number of adverse effects of antipsychotic medications. A previous study has shown that patients with MA use disorder are more likely to have extrapyramidal side-effects from antipsychotic medications (31). This study suggests that clinicians may have a better rationale for choosing to treat pMAP patients with lower doses of antipsychotics than SSD even before starting medication. For both pMAP patients and clinicians, having this rationale for optimizing treatment would be a great benefit. Future studies comparing pMAP and SSD, controlling for potential treatment-resistant psychosis factors, are needed. Additionally, suggesting the differences between pMAP and SSD, which are equally regarded in the DSM-5, may lead to a more subdivided and refined psychiatric diagnosis and treatment of heterogeneous “schizophrenia.” Further research on the differences found in this study is warranted.

## Data Availability Statement

The raw data supporting the conclusions of this article will be made available by the authors, without undue reservation.

## Ethics Statement

The studies involving human participants were reviewed and approved by the Clinical Research Ethics Board of the Medical Correction Center in East Japan. Written informed consent for participation was not required for this study in accordance with the national legislation and the institutional requirements.

## Author Contributions

YS designed the research protocol, collected the data, undertook data analysis, and wrote the initial draft of the paper. TO critically edited the paper. TO and YO reviewed the final draft of the paper. All authors contributed to the article and approved the submitted version.

## Conflict of Interest

The authors declare that the research was conducted in the absence of any commercial or financial relationships that could be construed as a potential conflict of interest.
